# Convergent Mechanistic Pathways Driving the Anaplastic Phenotype in Thyroid Cancer

**DOI:** 10.3390/ijms27167156

**Published:** 2026-08-10

**Authors:** Anthony Centone, Nicole R. DeSouza, Nan Yang, Shaun Desai, Augustine Moscatello, David Garber, Steven Hemmerdinger, Janine M. Rotsides, Mike Yao, Raj K. Tiwari, Jan Geliebter, Xiu-Min Li

**Affiliations:** 1 Department of Pathology, Microbiology & Immunology, New York Medical College, Valhalla, NY 10595, USA; acentone@nymc.edu (A.C.);; 2 Division of R&D, General Nutraceutical Technology, LLC., Briarcliff Manor, NY 10510, USA; 3 Department of Otolaryngology, New York Medical College, Valhalla, NY 10595, USA; 4 Department of Dermatology, New York Medical College, Valhalla, NY 10595, USA

**Keywords:** anaplastic thyroid cancer, tumor microenvironment, metabolic reprogramming, hypoxia signaling, ER stress

## Abstract

Anaplastic thyroid carcinoma (ATC) is a rare, highly aggressive follicular cell-derived malignancy characterized by rapid progression, profound dedifferentiation, and marked resistance to conventional therapy. Despite frequent involvement of major oncogenic pathways, ATC does not exhibit a universal driver mutation, suggesting that its pathogenesis reflects convergence upon shared biological hallmarks rather than dependence on a single molecular event. This review describes the principal mechanistic programs that define the ATC phenotype: disruption of cell-cycle and apoptotic control through alterations in *TP53*, *CDKN2A/B*, and aberrant MAPK activation; metabolic adaptations involving glycolysis, glutaminolysis, and mitochondrial one-carbon metabolism; reprogramming of canonical stress response pathways including ER stress and hypoxia signaling; and dynamic remodeling of the tumor microenvironment through cytokine-driven paracrine networks and immune modulation. Collectively, these processes cooperate to generate a highly proliferative, stress-tolerant, immune-inflamed yet immunosuppressed tumor state. A mechanistic understanding of these convergent pathways is essential for rational therapeutic development and for overcoming the profound clinical resistance that defines ATC.

## 1. Introduction

Anaplastic thyroid carcinoma (ATC) represents the most aggressive and lethal form of thyroid cancer. Arising from thyroid follicular cells, ATC is classified by its progressive loss of follicular cell differentiation and acquisition of a highly proliferative, invasive, and therapy-resistant phenotype. Although ATC accounts for a minority of thyroid cancer cases, its disproportionately high mortality underscores a critical need for a deeper understanding of its dynamic oncogenic nature. Unlike differentiated thyroid cancers, which often remain driven by core targetable oncogenic events, ATC lacks a single obligate driver mutation and instead reflects a convergence of genetic, metabolic, stress-adaptive, and microenvironmental alterations that collectively reinforce its aggressive state. Accordingly, ATC should be conceptualized not as the product of a singular linear pathway, but rather as a convergent endpoint reached through multiple distinct molecular and cellular actions. This review summarizes the current evidence and the core biological processes that define ATC, including loss of cell-cycle and apoptotic control, metabolic reprogramming, stress adaptation, and tumor microenvironment (TME) remodeling. It further examines how diverse molecular alterations converge to sustain these shared malignant programs.

### 1.1. Thyroid Structure and Function

The thyroid gland is a bilobular endocrine gland located in the anterior neck region, inferior to the larynx and anterior to the trachea [1]. This endocrine gland consists of two main cell types, follicular and parafollicular cells. As an endocrine organ, each of these cell types secretes different hormones central to mammalian physiology. Follicular cells are organized into spherical follicles enclosing a central lumen called the colloid, which is the space where the thyroid stores the glycoprotein thyroglobulin, a hormone precursor molecule synthesized and secreted by thyroid follicular cells. Parafollicular cells exclusively secrete calcitonin, a hormone involved in the regulation of calcium homeostasis [2].

The physiologically active thyroid hormones triiodothyronine (T3) and thyroxine (T4) are synthesized on a thyroglobulin backbone through iodine-dependent iodination and coupling reactions. Iodide is actively transported from the circulation into thyroid follicular cells via the sodium–iodide symporter and subsequently translocated into the colloid lumen. Within the colloid, thyroid peroxidase catalyzes the iodination and coupling of tyrosine residues on thyroglobulin, resulting in the formation of T3- and T4-containing thyroglobulin [3].

### 1.2. Anaplastic Thyroid Cancer

ATC belongs to a class of follicular cell-derived malignancies. Follicular cell cancers are categorized into two groups: differentiated and undifferentiated. Differentiated thyroid cancers include papillary thyroid cancer, follicular thyroid cancer, and Hurthle cell thyroid cancer [4]. Differentiated thyroid cancers consist of 95% of all thyroid cancers but have a significantly better prognosis than undifferentiated thyroid cancers [5].

ATC is an undifferentiated follicular cell thyroid cancer in which tumor cells exhibit profound loss of differentiation relative to their follicular cell origin [6]. ATC is extremely rare compared to other classes of thyroid cancer, representing less than 5% of all thyroid cancer cases [7]. However, ATC is highly aggressive, with a median survival of approximately 5 months [8]. Furthermore, ATC demonstrates marked resistance to conventional treatment modalities, underscoring a critical need for advanced mechanistic studies and the development of more effective therapeutic strategies [9].

## 2. Mechanisms of Anaplastic Thyroid Cancer

ATC is a highly heterogeneous malignancy that lacks a universal, obligate driver mutation, despite frequent involvement of major oncogenic pathways. Accordingly, this narrative review will first delineate the core biological processes that define the ATC phenotype, including: (1) Loss of cell cycle and apoptotic control, (2) metabolic adaptations, (3) stress adaptations, and (4) a polarizing TME. This review will subsequently examine the diverse genetic and biochemical alterations that converge to promote these shared processes. In this context, ATC should be viewed not as the product of a single linear pathogenic pathway, but rather as a phenotypically convergent end state that can be reached through multiple distinct molecular paths.

Although ATC may arise de novo in many cases, there is a large body of evidence that supports the biological progression of ATC through de-differentiation of pre-existing differentiated thyroid carcinomas [10]. In this context, early driver alterations such as *BRAF* and *RAS* mutations contribute to tumor initiation, while subsequent acquisition of additional genetic and epigenetic abnormalities promotes progressive loss of differentiation and emergence of the anaplastic phenotype [11]. Importantly, not all tumors follow the same evolutionary trajectory, and ATC remains a highly heterogeneous disease. Nevertheless, this progression model provides a useful conceptual framework for understanding how diverse molecular alterations may ultimately converge upon the shared biological programs that define ATC.

### 2.1. Loss of Cell Cycle and Apoptotic Control and Inflammatory Signaling

**Figure 1:**
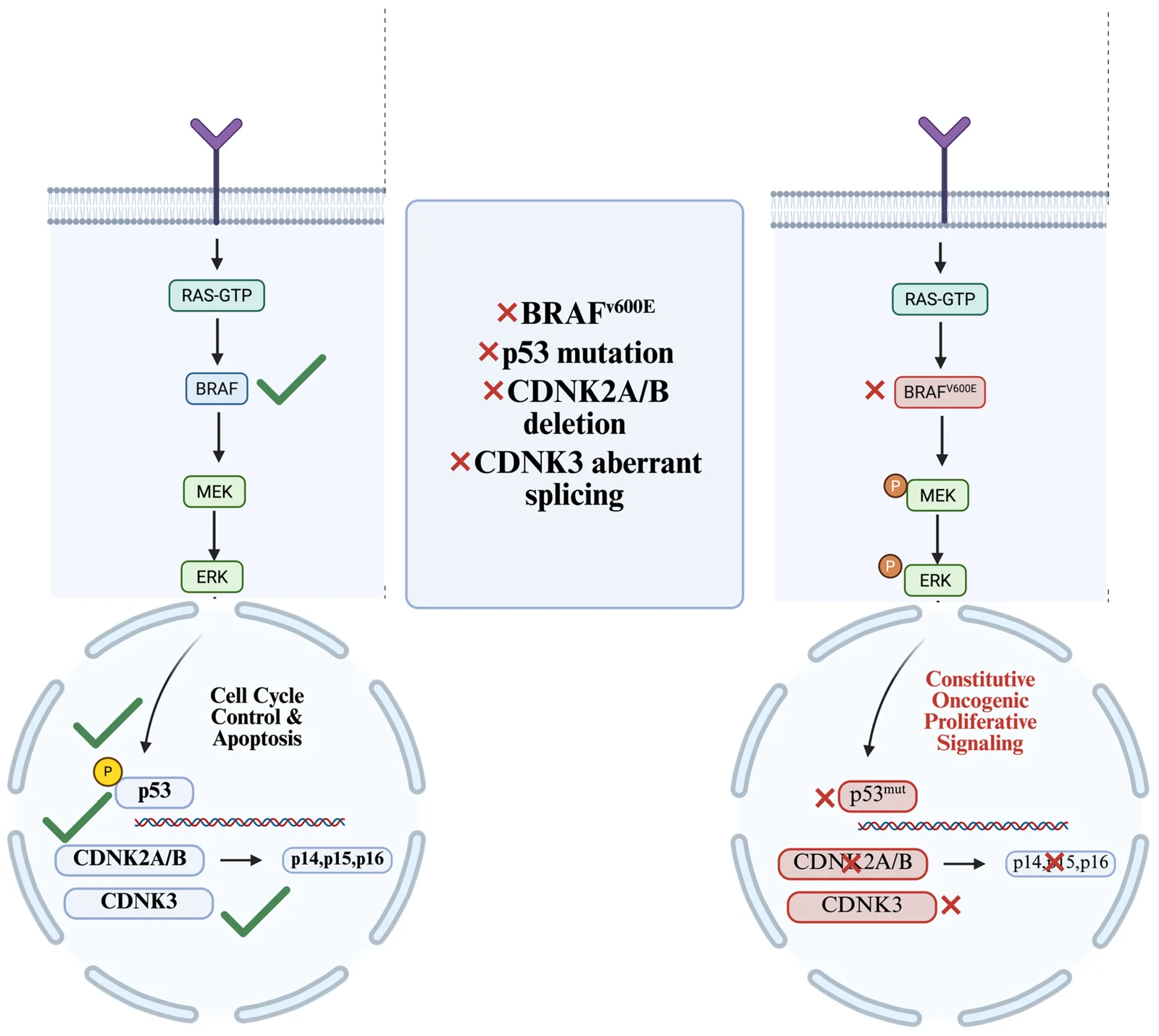
Loss of cell-cycle and apoptotic control in ATC. Schematic comparing regulated MAPK signaling and intact cell-cycle checkpoints in normal thyroid cells (**left**) with constitutive oncogenic signaling and checkpoint failure in ATC (**right**). In normal cells, receptor-mediated activation of RAS–BRAF–MEK–ERK signaling is tightly controlled and balanced by functional tumor suppressor pathways, including p53 and CDKN2A/B, which induce p14, p15, and p16 expression to restrain CDK activity and maintain cell-cycle arrest or apoptosis as needed. In ATC, activating *BRAF*^V600E^ mutations drive persistent MEK and ERK phosphorylation, resulting in constitutive proliferative signaling. Concurrently, *TP53* mutation, CDKN2A/B deletion, and aberrant *CDKN3* splicing disrupt critical G1/S checkpoint regulation and apoptotic surveillance mechanisms. The combined effect of sustained MAPK activation and loss of tumor suppressor function promotes unchecked cell-cycle progression and oncogenic proliferation characteristic of the anaplastic phenotype (Created in BioRender. Desouza, N. (2026) https://BioRender.com/4nqjxqi (accessed on 21 July 2026) and finalized with Microsoft PowerPoint).

The ability of a cancer cell to subvert cell cycle and apoptotic control is central to the unrestrained growth characteristics of a typical malignancy (Figure 1) [12].

The best characterized cell-cycle and apoptotic control gene involved in cancer is *TP53* [13]. While *TP53* gene mutations can be found in both differentiated thyroid cancer and ATC, their prevalence is much more common in ATC [14]. Loss of *TP53* function in ATC presumably allows for several other genetic driver events to proceed unabated, but possibly the most significant one is the interplay between itself and the BRAF gene. The *BRAF*^V600E^ mutation is commonly found in both well-differentiated thyroid cancers and ATC, playing a role in promoting cell proliferation, inducing MAPK signaling, and contributing to de-differentiation [15]. However, in well-differentiated thyroid cancers, the extent of damage this *BRAF*^V600E^ mutation can induce is limited by the failsafe system created by a functional *TP53* gene. In ATC cases where both *TP53* and *BRAF*^V600E^ mutations exist, the effects driven by *BRAF*^V600E^ are largely dysregulated, which contributes to the rapid proliferation and de-differentiation that is characteristic of ATC [16].

The *BRAF*^V600E^ mutation is a common oncogenic driver of many neoplasms, and is present in nearly one-third of ATC cases [17]. The *BRAF* gene is a central serine/threonine kinase involved canonically in the RAS/RAF/MEK/ERK (MAPK) signaling pathway [18]. The *BRAF*^V600E^ mutation is one that involves the substitution of a valine to a glutamic acid at codon number 600 (denoted V600E), permitting a constitutive “on” signal, leading to a strong neoplastic mitogenic signal through MAPK activation [19]. Targeting BRAF and MAPK signaling pathways in *BRAF*^V600E^ ATC variants is arguably the most effective ATC treatment available at this time.

Current standard-of-care targeted therapy for BRAF^V600E^ ATC variants consists of combined BRAF and MEK inhibition with dabrafenib and trametinib. This is often combined with PD-1 immune checkpoint inhibitor pembrolizumab, which respectively target mutant BRAF, downstream MEK1/2, and tumor-induced immune suppression. This regimen produces objective response rates of approximately 60% and extends median overall survival to approximately ~14–15 months, compared with historical survival of approximately ~5 months with conventional chemotherapy or chemoradiation. Emerging data suggest further improvements in progression-free and overall survival with the addition of immunotherapy as well [20]. It is important to note, however, that the *BRAF*^V600E^ mutation is not unique to ATC, as it is also present in a large proportion of papillary and other differentiated thyroid cancers [21]. Therefore, while *BRAF*^V600E^ renders a subset of ATCs targetable through MAPK-directed therapy, this alteration alone is insufficient to account for ATC transformation and does not serve as a discriminating factor between ATC and more differentiated thyroid malignancies. A summary of the principal therapeutic molecular targets discussed throughout this review, their corresponding therapeutic agents, and supporting references is provided in Table 1.

ATC has a strong propensity to subvert cell-cycle checkpoints and associated apoptotic responses, which facilitates its rapid, unrestrained growth. Several factors allow ATC cells to escape checkpoint-mediated growth arrest and consequently avoid apoptotic cell death. Transcriptomic analysis of surgically resected ATC tumors revealed a common signature of elevated expression of cell-cycle-associated genes, with cyclin-dependent kinase inhibitor 3 (*CDKN3*) among the most highly overexpressed. qRT-PCR and isoform-specific analysis demonstrated that this overexpression was largely attributable to an abnormally spliced *CDKN3* transcript predicted to lack normal inhibitory function, consistent with loss of effective CDK regulation and checkpoint control [25].

A large-scale screening collaboration project centered at the University of Colorado that sequenced 196 ATC tumors found that a high percentage of these tumors displayed deletions of the cyclin-dependent kinase inhibitor 2A (*CDKN2A*) and cyclin-dependent kinase inhibitor 2B (*CDKN2B*) genes [26]. *CDKN2A* and *CDKN2B* encode p14-, p15-, and p16-type tumor suppressor proteins that are central to governing cell-cycle control, specifically at the G1/S cell-cycle checkpoint [27].

Another parallel growth pathway discovered as an oncogenic driver in many ATC neoplasms is mTOR activation. Aberrant activation of the PI3K/Akt/mTOR signaling cascade is frequently observed in ATC and is associated with poor overall survival, highlighting this pathway as a central driver of proliferation, survival, and therapeutic resistance in ATC [27]. Preclinical studies targeting PI3K/mTOR signaling in ATC have demonstrated that pharmacologic inhibition of this axis, particularly in combination with CDK4/6 blockade, can synergistically suppress tumor cell proliferation and dramatically inhibit tumor growth in xenograft models. This supports the therapeutic potential of PI3K/mTOR pathway suppression to reverse key aspects of the aggressive ATC phenotype [28]. Additionally, natural compounds such as berberine have demonstrated anti-ATC activity in vitro by inhibiting proliferation and inducing both apoptosis and autophagic cell death in ATC cells through modulation of reactive oxygen species (ROS) generation and suppression of PI3K/AKT/mTOR signaling. This further highlights the therapeutic potential of targeting this pathway in ATC [22].

There is ample evidence supporting the role of aberrant JAK/STAT3 signaling contributing to the oncogenic nature of many ATC cancer stem cells (CSCs).

High-throughput functional screening in ATC models has identified JAK/STAT3 signaling as a critical regulator of stem-like tumorigenic properties, including sphere formation, anchorage-independent growth, and in vivo tumor progression. Pharmacologic inhibition of JAK/STAT3 and associated inflammatory pathways significantly impairs these stemness-associated behaviors, supporting a central role for IL-6-driven STAT3 activation in promoting ATC aggressiveness and therapeutic resistance [29].

Beyond its role in maintaining stem-like tumorigenic properties, JAK/STAT3 signaling exerts multifaceted oncogenic effects in tumorigenesis through both pro-oncogenic immune modulation and direct transcriptional activation of pro-growth signals. Persistent IL-6-mediated STAT3 activation triggers NF-κB to sustain a pro-inflammatory, immunosuppressive TME, reinforcing cytokine production and establishing feed-forward signaling loops that promote tumor progression [30]. STAT3 directly induces transcription of key proliferative regulators, including Cyclin D1 and c-Myc, thereby driving cell cycle progression, metabolic reprogramming, and resistance to apoptosis within ATC cells [31]. Through these combined extrinsic and intrinsic mechanisms, JAK/STAT3 signaling functions as a central integrator of inflammatory signaling and oncogenic growth control in ATC.

### 2.2. Metabolic Adaptations

**Figure 2:**
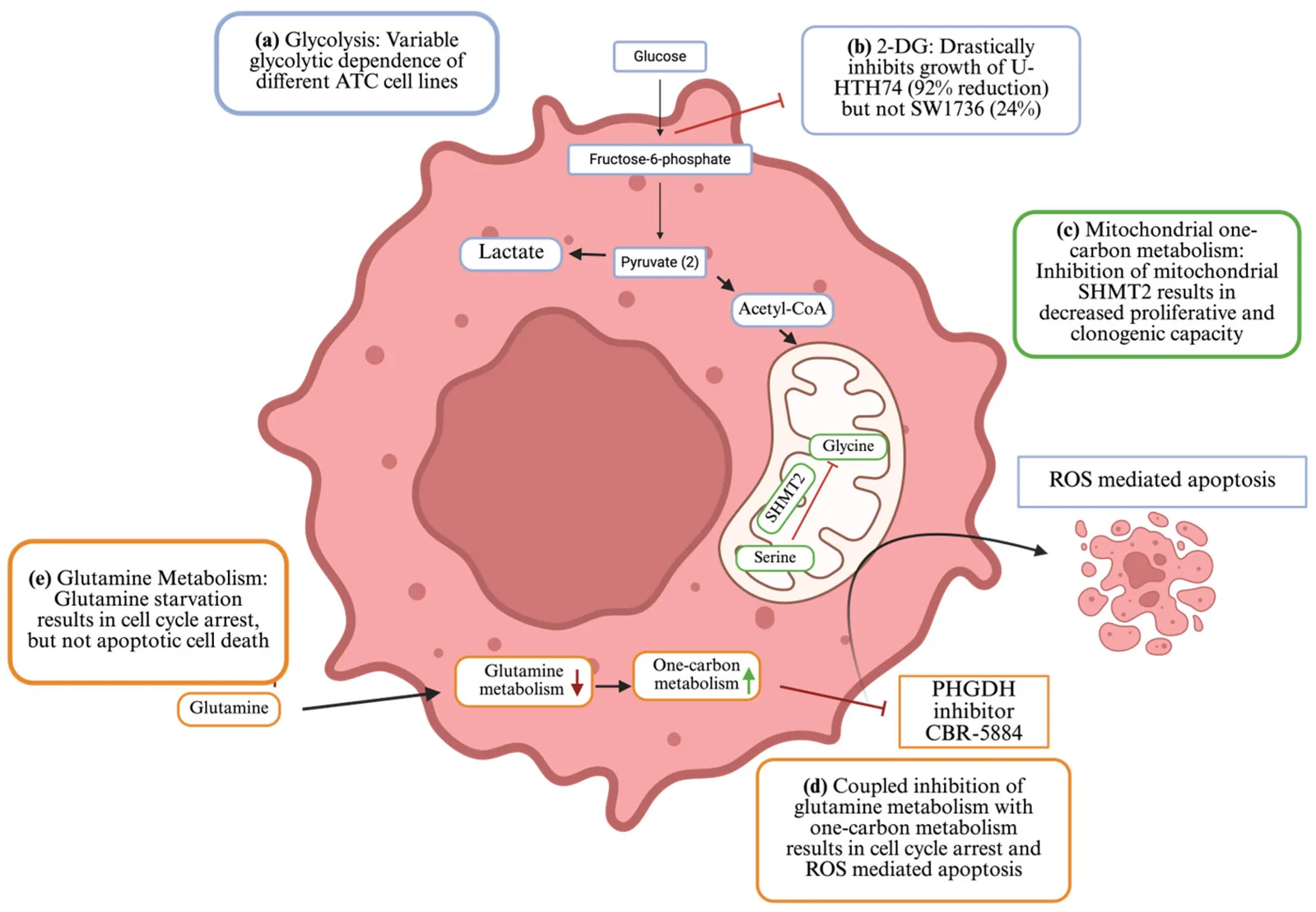
Metabolic adaptations supporting proliferation and stress tolerance in ATC. Schematic illustrating the major metabolic pathways described in ATC, highlighting variable dependence on glycolysis, glutamine metabolism, and mitochondrial one-carbon metabolism. (**a**) ATC cell lines exhibit heterogeneous reliance on glucose metabolism, with differential sensitivity to glycolytic inhibition; glucose-derived pyruvate contributes to lactate production or mitochondrial acetyl-CoA entry into the TCA cycle, supporting biosynthetic and energetic demands. (**b**) Treatment with 2-deoxyglucose (2-DG) markedly suppresses growth in U-HTH74 cells (~92% reduction) but produces more modest effects in SW1736 cells (~24% reduction), underscoring metabolic variability across models. (**c**) Mitochondrial one-carbon metabolism, mediated in part by serine hydroxymethyltransferase 2 (SHMT2), converts serine to glycine while generating one-carbon units that sustain nucleotide biosynthesis and redox homeostasis; inhibition of SHMT2 reduces proliferative and clonogenic capacity. (**d**) Coupled inhibition of glutamine metabolism and one-carbon metabolism promotes excessive ROS accumulation and triggers ROS-mediated apoptotic cell death. (**e**) Glutamine metabolism provides carbon intermediates that feed the TCA cycle and supports cytosolic and mitochondrial one-carbon pathways, with glutamine deprivation inducing cell-cycle arrest but not robust apoptotic cell death (red down arrow represents a decrease, green up arrow represents an increase). (Created in BioRender. Desouza, N. (2026) https://BioRender.com/22zdp9x (accessed on 21 July 2026) and finalized with Microsoft PowerPoint).

Metabolic adaptations are pivotal to cancer progression, as they are required to sustain the abnormally high rates of proliferation characteristic of malignant cells [32]. ATC cells exhibit a marked reliance on glucose metabolism for survival, and inhibition of glycolysis with 2-deoxyglucose, a glucose analog, increases the sensitivity of certain ATC cell lines to chemotherapy and radiation (Figure 2). Importantly, the degree of glucose dependence varies substantially among ATC models, with Sandulache et al. reporting a 92% reduction in growth under low-glucose conditions in the U-HTH74 cell line compared with only a 24% reduction in the SW1736 cell line [33].

Another emerging field of interest in ATC is one-carbon metabolism. One-carbon metabolism refers to folate-mediated one-carbon (methyl) transfer reactions that are central to an array of biosynthetic processes, including nucleotide, amino acid, and redox metabolism [34]. The intersection of glutamine metabolism and one-carbon metabolism has been shown to contribute significantly to metabolic adaptation and drug resistance in ATC. Inhibition of glutamine metabolism has been reported to induce cell-cycle arrest in certain ATC cell lines but fails to robustly promote apoptotic cell death [35]. A key study by Hwang et al. demonstrated that the ATC cell line 8505C upregulates one-carbon metabolism in response to glutaminolysis inhibition. They further showed that co-inhibition of glutamine metabolism (via glutamine deprivation) and one-carbon metabolism (using the PHGDH inhibitor CBR-5884) suppresses proliferative capacity while inducing excessive ROS accumulation, ultimately triggering ROS-dependent apoptotic cell death [36].

The most heavily studied metabolic contribution to ATC is mitochondrial metabolic reprogramming, particularly involving one-carbon metabolism. There is substantial evidence linking mitochondrial one-carbon metabolic pathways to ATC growth and survival. Serine hydroxymethyltransferase 2 (SHMT2) is a pivotal mitochondrial one-carbon metabolic enzyme that catalyzes the conversion of serine to glycine while transferring one-carbon units to folate cofactors, thereby supporting nucleotide biosynthesis and redox homeostasis [37]. RNA interference of the SHMT2 gene from cells of both human and mouse origin results in a significant reduction in proliferative capacity and clonogenic capacity [38]. SHMT2 overexpression can be used as both a predictive and prognostic biomarker of ATC [39].

### 2.3. Stress Adaptations

#### 2.3.1. Endoplasmic Reticulum Stress

**Figure 3:**
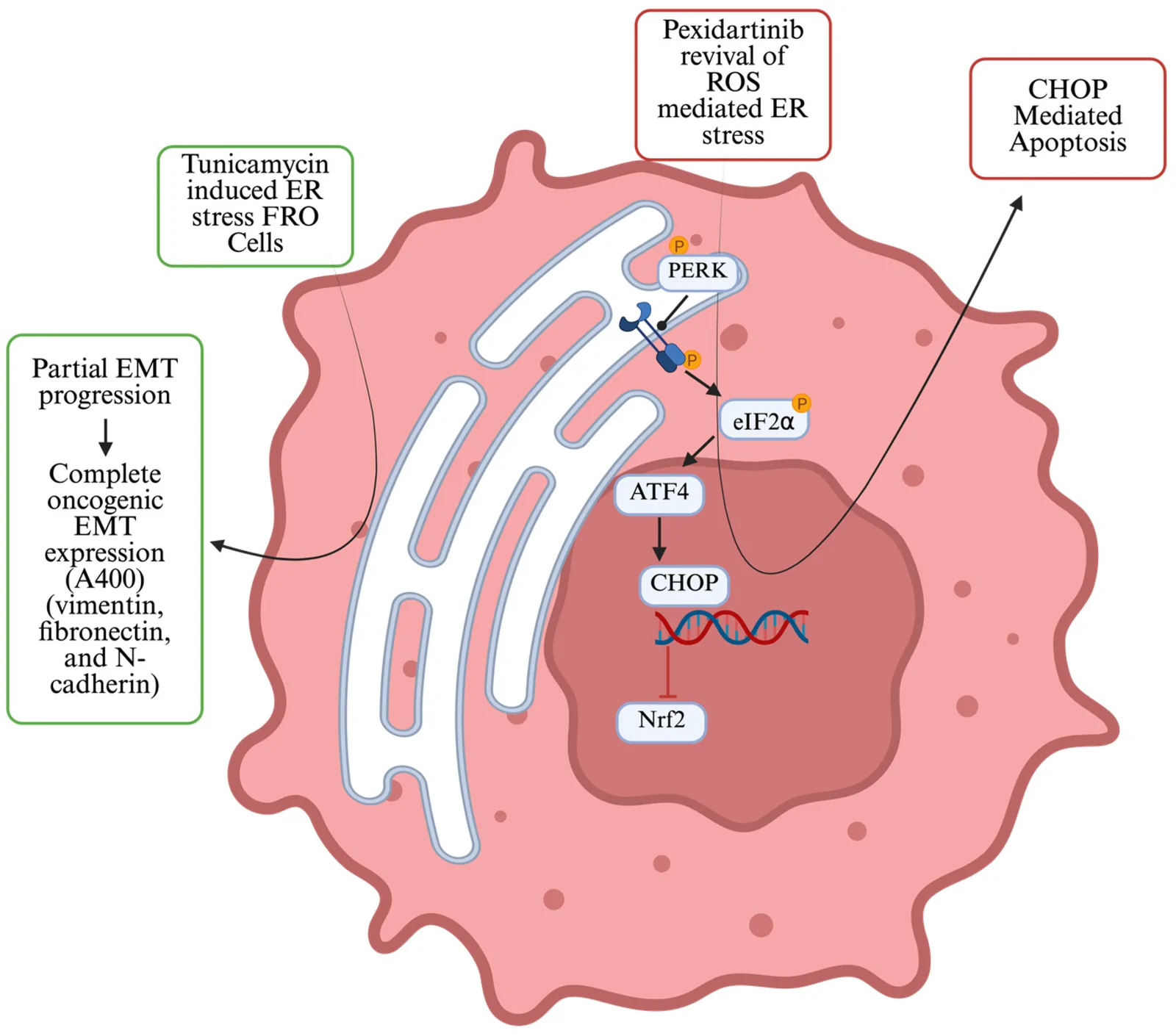
Divergent ER stress responses in ATC. Schematic depicting the dual roles of ER stress signaling in ATC, highlighting both adaptive, pro-tumorigenic reprogramming and cytotoxic stress responses. Tunicamycin-induced ER stress in FRO ATC cells activates the unfolded protein response (UPR), promoting partial epithelial-to-mesenchymal transition (EMT) and, with prolonged exposure, selection of a stress-adapted A400 subpopulation characterized by increased expression of mesenchymal markers including vimentin, fibronectin, and N-cadherin. In contrast, pharmacologic induction of ROS-amplified ER stress by pexidartinib activates the PERK–eIF2α–ATF4 axis, leading to CHOP induction and apoptotic cell death. This cytotoxic response is mechanistically linked to oxidative stress and involves modulation of Nrf2-dependent signaling pathways. Collectively, these findings illustrate that ATC cells can either reprogram canonical ER stress signaling toward adaptive EMT-promoting outputs or, under specific therapeutic conditions, engage terminal UPR pathways that culminate in CHOP-mediated apoptosis (Created in BioRender. Desouza, N. (2026) https://BioRender.com/mwp82km (accessed on 21 July 2026) and finalized with Microsoft PowerPoint).

Malignant progression is dependent on a tumor cell’s ability to sense, tolerate, and reprogram canonical cellular stress response pathways (Table 2) [40]. ATC can exploit a number of distinct cell stress pathways to sustain its highly aggressive phenotype (Figure 3). One of the best characterized stress adaptations in ATC is the subversion of endoplasmic reticulum (ER) stress signaling. Canonical ER stress mechanisms function as an intrinsic surveillance system that is activated by the accumulation of misfolded or unfolded proteins within the ER lumen, perturbations in calcium homeostasis, and increased secretory or metabolic demand, collectively engaging the unfolded protein response (UPR). Under normal physiological conditions, sustained activation of these pathways promotes translational attenuation, cell-cycle arrest, and, if homeostasis cannot be restored, the induction of apoptotic cell death [41]. Cancer cells find ways to grow either in spite of these stress pathways (by evading them) or with their help (by reprogramming them to facilitate pro-tumorigenic signaling) [42].

The ATC cell line FRO has been shown to undergo ER stress-dependent epithelial-to-mesenchymal transition (EMT). Lofrumento et al. described FRO cells as being “incompletely shifted” toward a mesenchymal phenotype, a shift that could be further driven by treatment with the ER stress-inducing compound tunicamycin. This EMT induction was so strong that the prolonged exposure to tunicamycin selected for a distinct, stress-adapted ATC subpopulation, termed A400. These tunicamycin-induced cells displayed increased expression of several mesenchymal markers, including vimentin, fibronectin, and N-cadherin, and exhibited enhanced invasive and migratory behavior in scratch-wound, adhesion, and invasion assays, consistent with a more mesenchymal and aggressive phenotype [43].

The previously described tunicamycin-induced ER stress is mediated by inhibition of N-linked glycosylation within the ER, which results in the accumulation of misfolded newly synthesized proteins and activation of the canonical UPR pathways [46]. The aforementioned study demonstrated the capacity of FRO ATC cells to reprogram this proteotoxic UPR signaling toward adaptive outputs that promote EMT and aggressive phenotypes. However, other forms of ER stress signaling may remain intact in ATC but are selectively engaged only under specific conditions. The multi-target tyrosine kinase inhibitor pexidartinib induces a distinct, ROS-amplified form of ER stress in ATC cells. In contrast to the pro-malignant effects associated with adaptive UPR activation, this ROS-mediated ER stress appears to activate a more cytotoxic stress response in the ATC cell lines CAL-62 and BHT101, resulting in growth inhibition and apoptosis. Mechanistically, the antitumor activity of pexidartinib was linked to activation of the PERK–eIF2α–ATF4–CHOP axis. It was shown to be dependent on Nuclear factor erythroid related factor 2 (Nrf2), as genetic or pharmacological suppression of Nrf2 attenuated ER stress signaling and significantly reduced the therapeutic efficacy of pexidartinib in these models [23].

#### 2.3.2. Hypoxia

Hypoxia is a major characteristic of many solid tumor cancers. As cancer tumors rapidly grow, the available blood supply in the TME loses its ability to perfuse the full tissue within, causing rapid metabolic shifts and induction of hypoxic stress signals [47]. The major transcriptional drivers of hypoxia-inducible pathways are Hypoxia-inducible factor (HIF) 1 and 2 [48].

In a large multi-institutional cohort study from China integrating both in-house patient samples and publicly available GEO datasets, transcript-based hypoxia scores were compared across thyroid cancer subtypes. Higher hypoxia scores were strongly associated with lower differentiation status, with ATC exhibiting the highest hypoxia scores among all thyroid cancer types. Consistent with this, ATC tumors also showed the highest expression of HIF-1α. Notably, HIF-1α levels positively correlated with PD-L1 transcript expression. Pharmacologic inhibition of HIF-1α signaling using the small-molecule inhibitor acriflavine reduced both PD-L1 and the oncogenic transcription factor c-MYC in ATC models [24]. Collectively, these findings indicate that ATC tumors do not merely tolerate hypoxic microenvironments, but actively exploit them by reprogramming hypoxia-responsive signaling pathways to drive oncogenic transcriptional programs and aggressive tumor phenotypes.

A closer examination of the mechanistic drivers linking hypoxia to oncogenic signaling in ATC helps connect hypoxia-responsive pathways with core drivers of the anaplastic phenotype. The histone demethylase KDM1A has been shown to demethylate HIF-1α at the protein level, thereby stabilizing it and preventing its proteasomal degradation [44]. Zhang et al. demonstrated that KDM1A is significantly overexpressed in ATC compared with normal thyroid tissue and papillary thyroid carcinoma, and that KDM1A-mediated stabilization of HIF-1α activates Wnt/β-catenin signaling, promoting stem-like transcriptional programs and EMT-associated phenotypes in ATC cells [45]. A summary of stress adaptation pathways discussed is presented in Table 2.

### 2.4. Genetic and Epigenetic Alterations

The preceding sections have described several of the major biological processes that define the malignant phenotype of ATC, including dysregulated proliferation, resistance to apoptosis, metabolic reprogramming, stress adaptation, and enhanced invasive potential. Many of these phenotypes arise as downstream consequences of recurrent genetic and epigenetic alterations (Table 3). Throughout this review, we have discussed the contributions of *TP53* mutation, *BRAF*V600E activation, RAS signaling, CDKN2A/CDKN2B deletion, aberrant CDKN3 splicing, and dysregulation of the PI3K/AKT/mTOR pathway to ATC progression. We have also highlighted the epigenetic regulator KDM1A, which promotes HIF-1α stabilization and activation of stemness-associated and epithelial-to-mesenchymal transition (EMT) transcriptional programs. While these alterations contribute substantially to the aggressive biology of ATC, several additional genetic and epigenetic events have also been implicated in disease progression and further illustrate the molecular complexity of this malignancy.

TERT promoter mutations are among the most common genetic alterations observed across a wide range of human malignancies and are frequently associated with aggressive disease behavior [49]. In thyroid cancer, TERT promoter mutations are commonly observed in ATC and are also associated with poor prognosis in patients with papillary thyroid cancer [50]. The TERT promoter regulates expression of telomerase reverse transcriptase (TERT), a critical component of the telomerase complex responsible for maintaining telomere length. Under normal physiological conditions, telomerase activity is limited in most somatic cells, resulting in progressive telomere shortening with each cell division. Once telomeres reach a critically short length, cells undergo senescence and permanently exit the cell cycle. TERT promoter mutations increase telomerase expression, allowing continued maintenance of telomere length and enabling cells to bypass senescence. This contributes to the uncontrolled proliferative capacity that is characteristic of malignancies such as anaplastic thyroid cancer [51].

Beyond its role in maintaining telomere length, TERT promoter mutations have also been implicated in thyroid cancer progression and dedifferentiation. Experimental studies have demonstrated that TERT cooperates with *BRAF*^V600E^ signaling to promote the transition from differentiated thyroid cancer toward more aggressive phenotypes. Mechanistically, TERT appears to enhance ribosomal biogenesis and protein synthesis programs, resulting in transcriptional reprogramming associated with loss of thyroid differentiation and acquisition of more malignant cellular characteristics [52].

In addition to alterations affecting telomere maintenance and MAPK signaling, activation of the PI3K/AKT/mTOR pathway is frequently observed in ATC through activating mutations in PIK3CA or loss of the tumor suppressor PTEN [53]. These alterations promote cell survival, proliferation, metabolic reprogramming, and resistance to apoptosis, contributing to the highly aggressive phenotype characteristic of ATC. Furthermore, aberrant PI3K/AKT/mTOR signaling has emerged as a potential therapeutic target and may contribute to resistance against conventional treatment approaches [28].

Epigenetic dysregulation has also emerged as an important contributor to ATC progression. As discussed previously, overexpression of the histone demethylase KDM1A promotes stabilization of HIF-1α, activation of Wnt/β-catenin signaling, and induction of EMT-associated transcriptional programs. Beyond its effects on specific signaling pathways, KDM1A highlights the broader role of epigenetic regulation in ATC, where alterations in chromatin structure and transcriptional control can promote dedifferentiation, stem-like phenotypes, and increased invasive capacity [45]. These findings suggest that epigenetic mechanisms may complement genetic alterations in driving the aggressive behavior characteristic of ATC.

**Table 3:** Genetic and epigenetic drivers of ATC.

Alteration	Type	Functional Consequence	Contribution to ATC Malignancy	Ref.
*TP53* mutation	Genetic	Loss of cell-cycle checkpoint control and apoptotic signaling	Enhanced survival, genomic instability, uncontrolled proliferation	[14,16]
*BRAFV600E* mutation	Genetic	Constitutive MAPK pathway activation	Increased proliferation, dedifferentiation, tumor progression	[15,16,17,18,19,20,21]
*RAS* mutation	Genetic	Activation of MAPK and PI3K signaling pathways	Enhanced proliferation, survival, and metastatic potential	[11]
*CDKN2A/CDKN2B* deletion	Genetic	Loss of G1/S cell-cycle regulation	Increased cell-cycle progression and proliferation	[26,54]
Aberrant CDKN3 splicing	Genetic	Impaired CDK regulation	Dysregulated cell-cycle progression	[25]
TERT promoter mutation	Genetic	Increased telomerase expression and maintenance of telomere length; Enhanced ribosomal biogenesis and transcriptional reprogramming	Escape from senescence, dedifferentiation, tumor progression; Dedifferentiation and progression toward aggressive phenotypes	[50,51,52]
PIK3CA mutation	Genetic	Activation of PI3K/AKT/mTOR signaling	Enhanced proliferation, metabolism, and survival	[27,28,53]
KDM1A overexpression	Epigenetic	Histone demethylation, HIF-1α stabilization, activation of Wnt/β-catenin signaling	EMT, stemness, invasion, and dedifferentiation	[44,45]

### 2.5. Tumor Microenvironment

**Figure 4:**
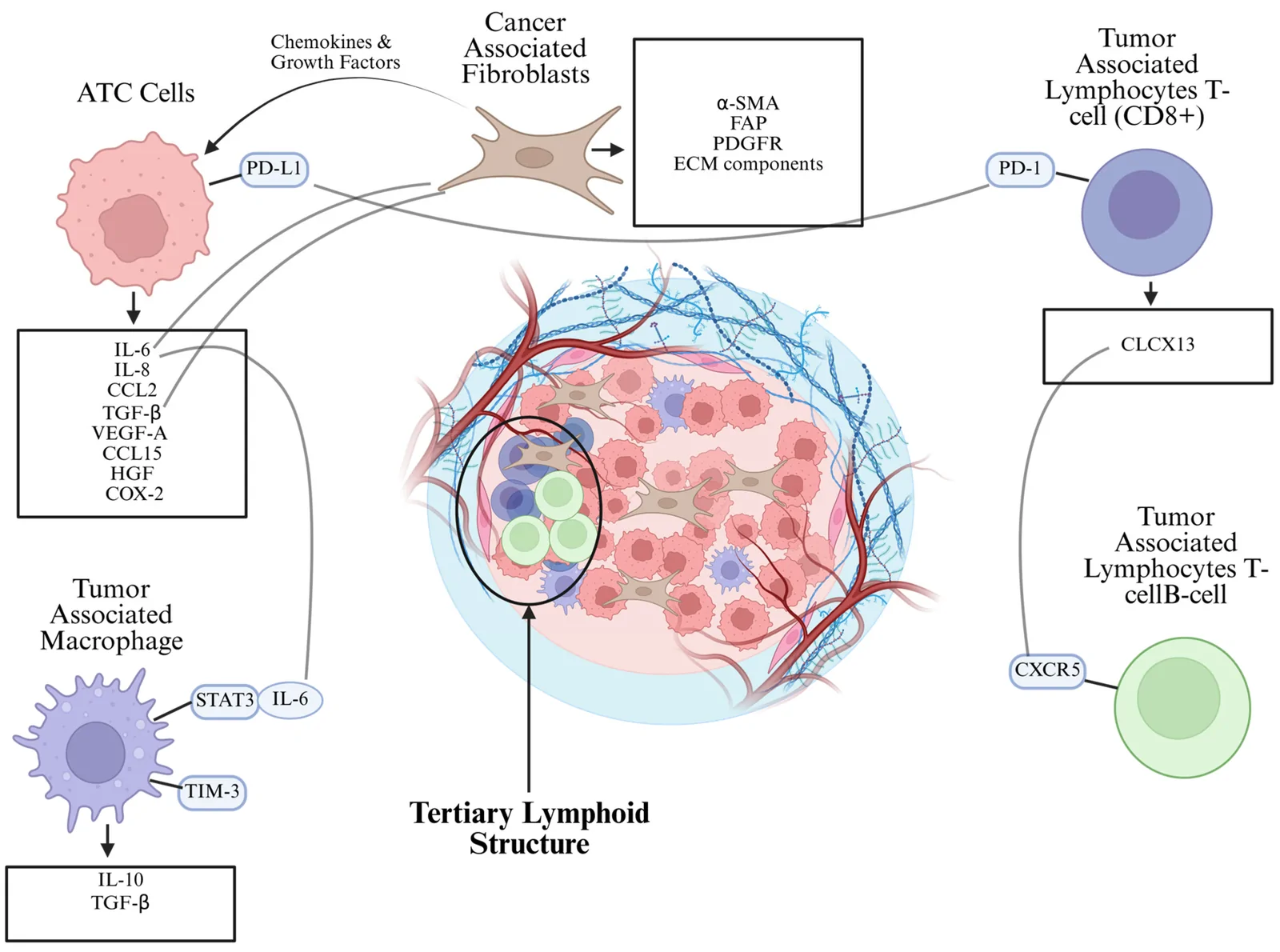
Immune-inflamed yet immunosuppressive TME in ATC. Schematic illustrating the complex cellular and cytokine networks that define the ATC TME. ATC tumor cells secrete high levels of IL-6, which activates STAT3 signaling in surrounding immune cells and reinforces chronic inflammatory yet tumor-permissive signaling loops. Tumor-derived factors promote recruitment and polarization of tumor-associated macrophages (TAMs) toward an M2-like phenotype, characterized in part by TIM-3 expression and immunosuppressive cytokine production. Concurrently, ATC cells express PD-L1, which engages PD-1 on infiltrating CD8^+^ T cells and contributes to T-cell exhaustion and impaired anti-tumor immunity. The TME also contains CXCL13^+^ T cells and CXCR5^+^ B cells associated with tertiary lymphoid structure-like immune niches, reflecting the paradoxical immune-inflamed state of ATC. Despite dense immune infiltration, coordinated PD-1/PD-L1 signaling, macrophage reprogramming, and persistent IL-6/STAT3 activation collectively sustain immune dysfunction while promoting tumor progression. This model highlights how ATC tumors actively shape an immunologically “hot” yet functionally immunosuppressed microenvironment that contributes to therapeutic resistance (Created in BioRender. Desouza, N. (2026) https://BioRender.com/l7owqzg (accessed on 21 July 2026) and finalized with Microsoft PowerPoint).

The TME refers to the network of non-malignant cells, extracellular matrix, vasculature, immune cells, and biochemical conditions that surround and infiltrate a tumor and collectively regulate tumor growth, invasion, immune evasion, metabolic adaptation, and therapeutic response (Figure 4) [55].

#### 2.5.1. ATC Paracrine Signaling

Before describing the intricate network of cellular components that comprise the TME, it is important to first consider the influence exerted by ATC cells themselves. ATC cells secrete multiple cytokines and soluble mediators that actively shape and sustain a cancer-permissive microenvironment. Among these, ATC cells classically express high levels of IL-6, a potent activator of STAT3 that modulates the functional programming of several cell types within the ATC TME (Table 3) [56]. In parallel, ATC cells secrete the pro-oncogenic chemokines IL-8 and CCL2, which promote macrophage recruitment and angiogenesis. Accordingly, suppression of these mediators in ATC cells has been shown to reduce macrophage infiltration and vascularization [57]. Once recruited, these macrophages are further reprogrammed by ATC-derived TGF-β, reinforcing an immune-permissive environment that supports tumor progression [51]. Beyond these key pathways, ATC cells continue to modulate TME composition and function through additional mediators, including VEGF-A, CCL15, HGF, and COX-2 [58].

#### 2.5.2. Immune Response

Cancerous tumors are often described along a spectrum of “immunologically hot” versus “immunologically cold” phenotypes. Immunologically hot tumors are characterized by a prominent inflammatory transcriptional signature and dense infiltration of immune cells, whereas immunologically cold tumors exhibit sparse immune cell infiltration and weak inflammatory or interferon signaling [59]. In general, immune-inflamed (“hot”) tumors are more likely to respond to immunotherapeutic interventions, particularly immune checkpoint blockade, than their immune-cold counterparts, making characterization of tumor immune states a central focus for improving outcomes in treatment-refractory malignancies [60].

ATC represents a paradox among solid tumors in that its TME is characteristically immune-inflamed yet remains largely refractory to durable responses from immunotherapeutic interventions [61]. This disconnect suggests that, despite abundant immune infiltration, potent local immunosuppressive and tumor-promoting programs dominate within the ATC TME. Accordingly, describing how ATC tumors maintain immune dysfunction and evade effective anti-tumor immunity within an otherwise immunologically active environment is of central importance for understanding therapeutic resistance and for discovering effective treatment strategies.

#### 2.5.3. Hallmark Immune ATC Mediators

The TME of ATC consists of a dynamic combination of immune-stimulatory and immune-suppressive cytokines and soluble mediators. Prominent among these are IL-6, TGF-β, interferon-induced chemokines (CXCL9, CXCL10, CXCL8, and CXCL11), CCL2, CSF-1, and VEGF, which are hallmarks of the ATC TME and collectively orchestrate a complex, pro-tumorigenic, and immunoregulatory environment [62,63,64].

ATC cells also secrete elevated levels of TGF-β. Suppression of TGF-β signaling in ATC cell lines using silencing RNA has been shown to reverse malignant phenotypes such as invasion and migration [65]. TGF-β signaling operates in both autocrine and paracrine fashions, influencing ATC cells directly as well as neighboring stromal and immune populations. In this context, TGF-β has been shown to play a major role in macrophage polarization and cancer-associated fibroblast (CAF) modulation within the ATC TME [66,67]. Beyond IL-6 and TGF-β, ATC cells also secrete VEGF, CXCL8/IL-8, and CCL2, each of which has been implicated in ATC tumor progression through pro-angiogenic and immune-modulatory remodeling of the TME [57,68].

#### 2.5.4. Tumor-Associated Macrophages

Tumor-associated macrophages (TAMs) play a significant role in the survival and progression of ATC. The ATC TME is characterized by dense infiltration of alternatively activated, M2-polarized macrophages, which exhibit immunosuppressive and tumor-promoting functions and are thought to contribute to the highly aggressive phenotype of this disease [69]. At first glance, this M2-dominant macrophage landscape appears paradoxical given the strongly immune-inflamed nature of the ATC microenvironment.

Insight into this phenomenon is provided by Lu et al., which employed single-cell RNA sequencing and pseudo-temporal trajectory analysis of clinical thyroid cancer specimens to infer dynamic changes in the different cellular compartments during ATC cell transformation. Their analysis suggested that macrophage polarization during early transformation exhibits pro-inflammatory, interferon-responsive, M1-like transcriptional signatures, consistent with strong paracrine inflammatory signaling from the ATC cells. In contrast, at later pseudo-temporal stages corresponding to advanced ATC, macrophages acquire immunosuppressive, tissue-remodeling, M2-like programs that coincide with the emergence of T-cell exhaustion, indicating coordinated reprogramming of the immune microenvironment toward tumor-promoting states [70].

Importantly, this study did not track macrophage polarization in real time, but rather inferred cellular state transitions in silico based on transcriptional similarity and tissue context. Nonetheless, these findings support a model in which early inflammatory activation of macrophages gives way to dominant M2-like, immunosuppressive programs during ATC progression, highlighting a central paradox of an immune-inflamed but also immunosuppressed TME.

While the exact progression of macrophage polarization in ATC remains to be fully elucidated, substantial evidence supports specific molecular mechanisms that reinforce the ultimately dominant M2-like fate of tumor-associated macrophages. Using conditioned media from human ATC cell lines (8505c and KTC-2) to model tumor-educated macrophages in vitro, Stempin et al. identified a strong association between M2 polarization and surface expression of the immune checkpoint receptor T-cell immunoglobulin and mucin-domain containing-3 (TIM-3). THP-1–derived macrophages exposed to ATC-conditioned media upregulated TIM-3 and acquired an M2-like phenotype, and antibody-mediated TIM-3 inhibition partially reversed this polarization, as evidenced by reduced expression of canonical M2 markers CD206 and CD163 and decreased secretion of immunosuppressive cytokines including TGF-β and IL-10. In parallel, ATC-derived soluble factors strongly activate the IL-6/STAT3 signaling axis in tumor-educated macrophages, a pathway known to promote chronic inflammatory yet immunosuppressive programs and to further stabilize M2-like polarization within the ATC TME [71].

#### 2.5.5. Tumor Infiltrating Lymphocytes

Like many other solid tumors, the TME of ATC encapsulates a large population of exhausted lymphocytes, particularly exhausted CD8^+^ T cells [72]. Single-cell transcriptomic profiling has revealed that ATC is frequently associated with a high abundance of functionally exhausted CD8^+^ T cells and elevated expression of inhibitory checkpoint molecules, including PD-1/PD-L1, which reflects adaptive immune dysfunction in ATC [70]. Furthermore, this exhausted CD8^+^ T-cell phenotype appears substantially more frequently in ATC than in most differentiated thyroid cancers, highlighting T-cell exhaustion as a key differentiator between ATC and differentiated thyroid cancers [73]. PD-1/PD-L1 blockade has been reported to prolong survival in a subset of ATC patients. However, studies have yielded mixed results regarding whether tumor PD-L1 expression levels reliably correlate with therapeutic responsiveness, indicating that the exact role of PD-1/PD-L1 in ATC T-cell exhaustion needs to be further studied [74,75].

While exhausted T cells are largely immune-quiescent relative to their fully effector-competent counterparts, they still retain selective functional programs that can contribute to anti-tumor immune activity, particularly through the production of chemokines and other signaling molecules that promote immune cell recruitment within the TME. Single-cell RNA-sequencing analyses comparing papillary thyroid cancer and ATC tumor samples have revealed a distinct subset of CXCL13^+^ T cells that are markedly enriched in ATC tumors relative to papillary thyroid cancers. These CXCL13^+^ T cells are associated with the formation of tertiary lymphoid structure-like immune clusters, which may represent localized, immunologically active niches within an otherwise immune-exhausted environment [76]. This organization may help explain why ATC tumors frequently present as immunologically “hot” while simultaneously fostering profound T-cell dysfunction. Although accumulating evidence links CXCL13^+^ T cells to responsiveness to PD-1/PD-L1 checkpoint blockade across multiple cancer types, including ATC, there remains a notable lack of mechanistic studies establishing direct causality or defining the precise functional role of this population beyond the presumed canonical recruitment of CXCR5^+^ B cells [77,78].

#### 2.5.6. Cancer Associated Fibroblasts

CAFs represent a critical stromal component of the ATC TME, where they function as dynamic regulators of extracellular matrix (ECM) remodeling, paracrine signaling, and immune modulation (Table 4). In thyroid malignancies, fibroblasts are actively reprogrammed by ATC tumor-derived mediators, including TGF-β, IL-6, and other inflammatory cytokines, into persistent, activated phenotypes characterized by increased expression of α-SMA, FAP, PDGFR, and ECM components [67,79]. In ATC specifically, bidirectional signaling between tumor cells and fibroblasts enhances pro-tumorigenic behavior, as demonstrated by co-culture models showing that ATC cells “educate” fibroblasts to adopt CAF-like phenotypes that subsequently promote tumor cell proliferation, migration, and invasion [80]. Beyond structural remodeling, CAFs contribute to the inflammatory landscape of ATC through secretion of chemokines and growth factors that reinforce STAT3- and TGF-β-dependent signaling pathways, thereby supporting tumor progression and resistance to therapy [58,67]. Recent single-cell transcriptomic analyses further highlight the heterogeneity of fibroblast populations in aggressive thyroid cancers, identifying distinct inflammatory and myofibroblastic CAF programs that spatially localize to invasive fronts and dedifferentiated tumor regions, suggesting that specific CAF subsets may differentially contribute to ATC pathogenesis [70]. Collectively, these findings position CAFs not merely as passive structural elements, but as active orchestrators of ATC progression through coordinated ECM remodeling, paracrine amplification loops, and immune shaping within the TME.

## 3. Conclusions

**Figure 5:**
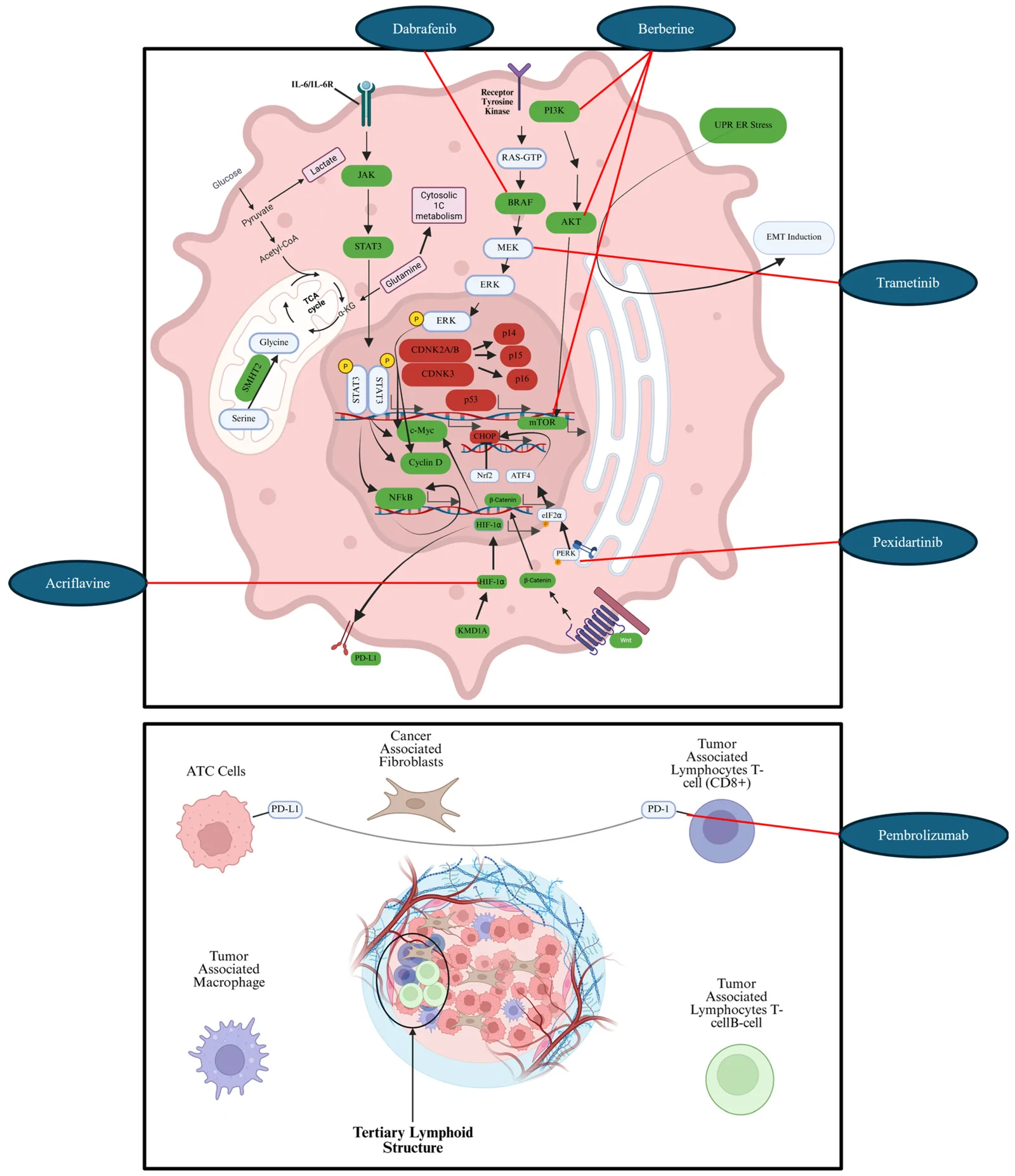
Integrated schematic of dysregulated signaling pathways and therapeutic intervention points in ATC. Comprehensive overview of the major intracellular signaling pathways, metabolic adaptations, stress-response mechanisms, and TME interactions discussed throughout this review that collectively contribute to the aggressive phenotype of ATC. The diagram illustrates how multiple oncogenic signaling cascades converge to promote sustained proliferation, resistance to apoptosis, metabolic reprogramming, and immune evasion within ATC cells. Proteins described in the literature as mutated or functionally inactivated tumor suppressors are highlighted in red, whereas signaling components characterized by constitutive activation or oncogenic signaling are highlighted in green, illustrating the shift from tightly regulated cellular signaling to the dysregulated molecular landscape characteristic of ATC. In addition to these dysregulated nodes, the figure highlights clinically relevant therapeutic intervention points, with pharmacologic agents shown in blue labels outside the schematic and connected to their corresponding molecular targets within the signaling network. Collectively, this integrated schematic illustrates how diverse molecular alterations cooperate to drive ATC pathogenesis while also highlighting cellular targets that may be exploited for therapeutic intervention (Created in BioRender. Desouza, N. (2026) https://BioRender.com/j3i9s4g (accessed on 21 July 2026) and finalized with Microsoft PowerPoint).

ATC emerges as a biologically convergent malignancy defined not by a singular oncogenic mutation, but by the coordinated disruption of regulatory systems governing proliferation, metabolism, stress tolerance, and immune homeostasis. Loss of cell-cycle checkpoint control, constitutive activation of mitogenic and inflammatory signaling pathways, metabolic rewiring to sustain biosynthetic and redox demands, adaptive exploitation of ER stress and hypoxia pathways, and dynamic remodeling of the TME collectively reinforce the ATC phenotype. Importantly, ATC illustrates the paradox of an immune-inflamed yet functionally immunosuppressed tumor ecosystem, highlighting the complexity of therapeutic intervention in this disease. Future progress will depend on integrating genomic, transcriptomic, metabolic, and microenvironmental insights to identify combination strategies capable of simultaneously targeting multiple reinforcing malignant circuits (Figure 5). Understanding ATC as a phenotypically convergent endpoint provides a conceptual framework for designing rational, multi-axis therapeutic approaches aimed at disrupting the coordinated networks that sustain this highly aggressive cancer.

## Figures and Tables

**Table 1:** Therapeutic Targets.

Drug Name	Therapeutic Target	Ref.
Dabrafenib	BRAF	[20]
Trametinib	MEK	[20]
Berberine	PI3K/AKT/mTOR	[22]
Pexidartinab	PERK	[23]
Acriflavine	HIF-1α	[24]
Pembrolizumab	PD-1	[20]

**Table 2:** Stress adaptation pathways in ATC.

Stress Type	Primary Molecular Drivers/Pathway Components	Experimental Model(s) Referenced	Functional Outcome in ATC	Ref.
ER Stress—Adaptive/EMT-promoting	Tunicamycin → UPR activation; ER stress–dependent EMT; mesenchymal markers (vimentin, fibronectin, N-cadherin)	FRO cells; A400 subpopulation	Enhanced EMT, migration, invasion; selection of stress-adapted aggressive phenotype	[43]
ER Stress—ROS-amplified Cytotoxic Response	Pexidartinib → PERK–eIF2α–ATF4–CHOP axis activation; Nrf2-dependent signaling	CAL-62, BHT101	Growth inhibition; apoptosis	[23]
Hypoxia Signaling	HIF-1α overexpression; hypoxia transcript scores	Clinical ATC cohorts; GEO datasets	Dedifferentiation; oncogenic transcriptional reprogramming	[24]
Hypoxia–Immune Axis	HIF-1α → PD-L1 and c-MYC regulation; inhibition with acriflavine	ATC models	Reduction of PD-L1 and c-MYC; modulation of immune evasion	[24]
Epigenetic Stabilization of Hypoxia Signaling	KDM1A-mediated HIF-1α stabilization; Wnt/β-catenin activation	ATC cells (comparative expression studies)	Stem-like transcriptional programs; EMT phenotypes	[44,45]

**Table 4:** TME cellular components and relative secreted mediators.

Cell Type	Mediators Secreted
ATC Tumor Cell	IL-6; TGF-β; VEGF; CXCL8 (IL-8); CCL2
Tumor-Associated Macrophages (TAMs)	TGF-β; IL-10 (associated with M2-like macrophages exposed to ATC-conditioned media)
Tumor-Infiltrating Lymphocytes (Exhausted CD8^+^ T cells)	CXCL13
Cancer-Associated Fibroblasts (CAFs)	Chemokines; Growth factors

## Data Availability

No new data were created or analyzed in this study. Data sharing is not applicable to this article.

## Abbreviations

ATC	Anaplastic Thyroid Carcinoma
CAF	Cancer associated fibroblast
CDKN2A	Cyclin-dependent kinase inhibitor 2A
CDKN2B	Cyclin-dependent kinase inhibitor 2B
CDNK3	Cyclin-dependent kinase inhibitor 3
CSC	Cancer stem cells
ECM	Extracellular matrix
EMT	Epithelial-to-mesenchymal transition
HIF	Hypoxia-inducible factor
Nrf2	Nuclear factor erythroid related factor 2
ROS	Reactive oxygen species
SHMT2	Serine hydroxymethyltransferase 2
T3	Triiodothyronine
T4	Thyroxine
TAMs	Tumor associated macrophage
TIM-3	T-cell immunoglobulin and mucin-domain containing-3
UPR	Unfolded protein response
